# Supplementary material for: Loss of Zfp335 triggers cGAS/STING-dependent apoptosis of post-β selection thymocytes
Source: Nat Commun. 2022 Oct 6;13:5901. doi: 10.1038/s41467-022-33610-4 (PMC9537144; doi:10.1038/s41467-022-33610-4)
Supplement: Supplementary file 1 — Supplementary Information [file 41467_2022_33610_MOESM1_ESM.pdf]

Supplementary Information

Loss of Zfp335 triggers cGAS/STING-dependent apoptosis of post-β selection thymocytes

Jeremy J Ratiu[1*], William E Barclay[1], Elliot Lin[1], Qun Wang[1], Sebastian Wellford[1], Naren Mehta[1], Melissa J Harnois[1], Devon DiPalma[1], Sumedha Roy[1], Alejandra V Contreras[2], Mari L Shinohara[1,3], David Wiest[2], Yuan Zhuang[1]

*****Corresponding Author: Dr. Jeremy Ratiu, email: Jeremy.Ratiu@duke.edu

This PDF file includes:

Supplementary Figures 1-12

Supplementary Tables 1-2

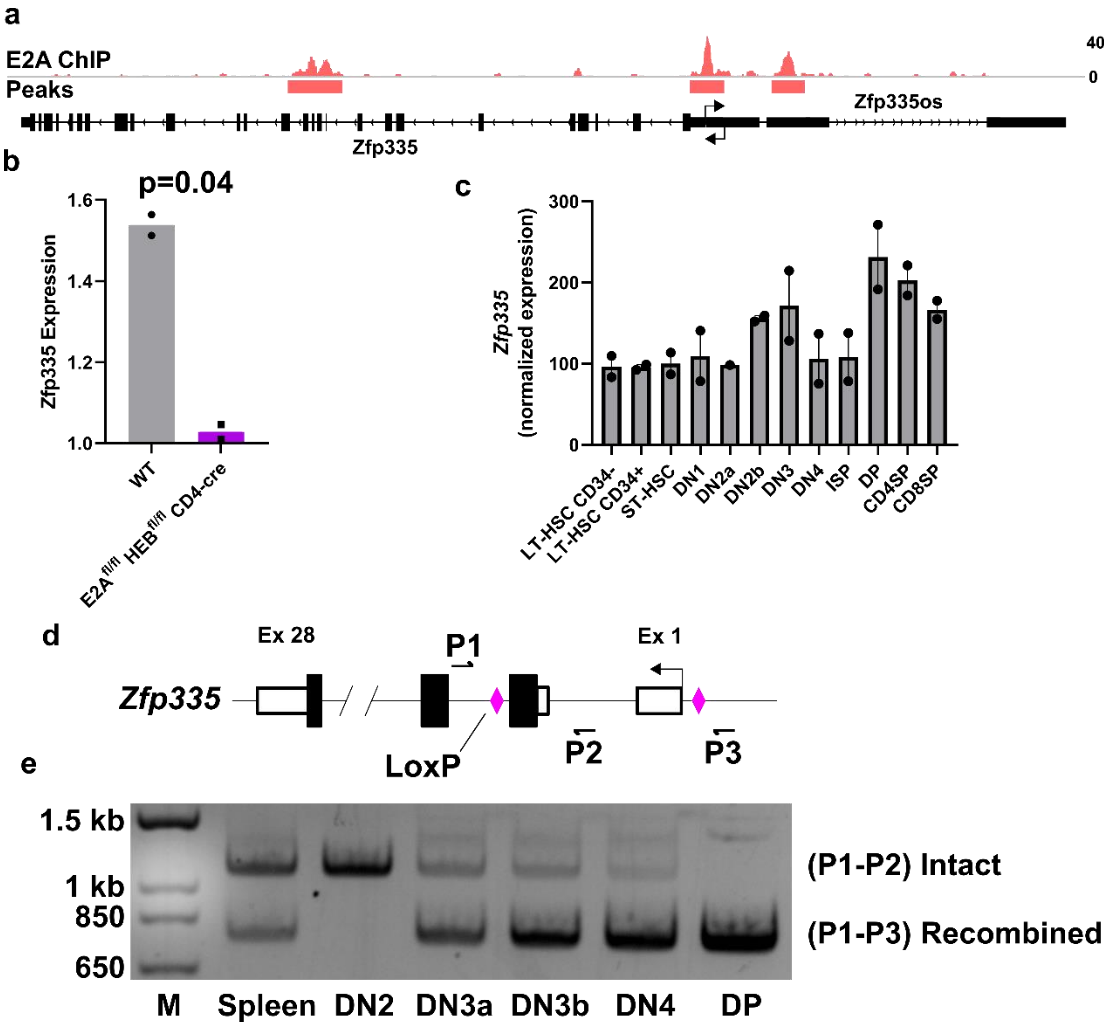

Supplementary Figure 1 – Zfp335 is a target of E proteins in developing T cells. (a) E2A ChIP-seq track for *Zfp335* locus in *Id2*$^{fl/fl}$ *Id3*$^{fl/fl}$ *Lck-cre* DP thymocytes (GSE89849). (b) Zfp335 transcript abundance in WT vs. E2A/HEB double knock-out DP thymocytes determine by microarray (GSE9749, n=2 each). (c) Zfp335 expression throughout T cell development determined by RNA-seq (GSE109125). (d) Schematic diagram for PCR-based determination of Zfp335 recombination kinetics. Small arrows indicate approximate positions for primers (P1-3) used for assay. (e) Representative assessment of Zfp335 recombination in sort purified *Zfp335*$^{fl/fl}$ *E8*$_{III}$*-cre* spleen, DN2, DN3a, DN3b, DN4 or DP thymocytes. M is 1kb plus DNA ladder. Data are representative of four individual experiments (e). Data points indicate biological replicates (b-c). Source data are provided as a Source Data file.

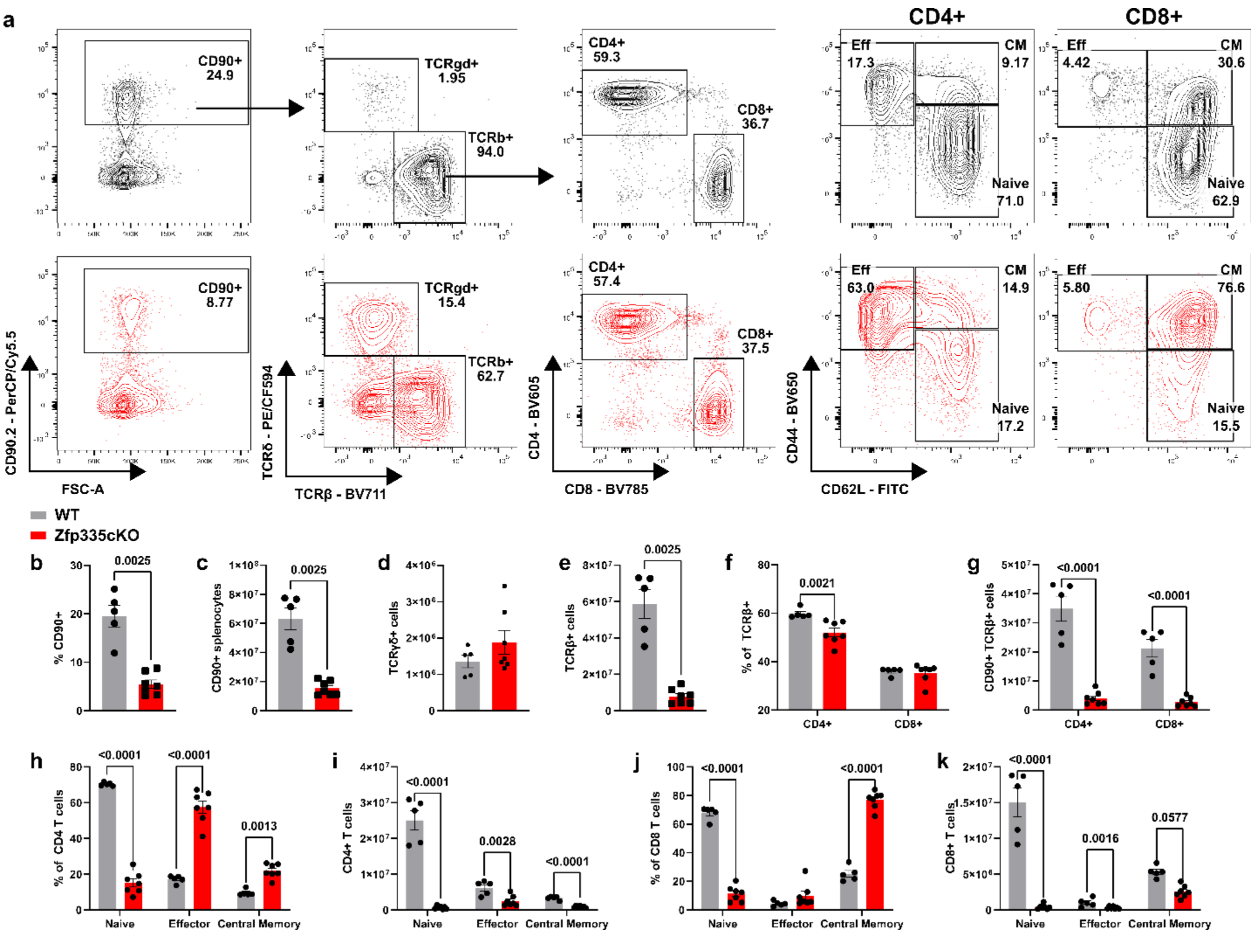

Supplementary Figure 2 – Zfp335cKO mice exhibit T lymphopenia and reduced peripheral naïve T cells. (a) Gating schema for identification of WT (black) or Zfp335cKO (red) splenic T cell populations beginning with live (DAPI⁻) splenocytes. Proportion (b) or total numbers (c) of splenic CD90+ cells. Total numbers of TCRγδ+ (d) or TCRαβ+ (e). Proportions (f) and total numbers of CD4+ or CD8+ TCRαβ cells. Proportions (h,j) and numbers (i,k) of naïve, effector or central memory T cells within the CD4+ or CD8+ compartment. WT (n=5) or Zfp335cKO (n=7) male and female mice from two separate experiments. *P*-values determined by two-tailed Mann-Whitney U-test (b-e) or Two-Way ANOVA with *post hoc* Sidak test (f-k). Plots show mean ± sem. Source data are provided as a Source Data file.

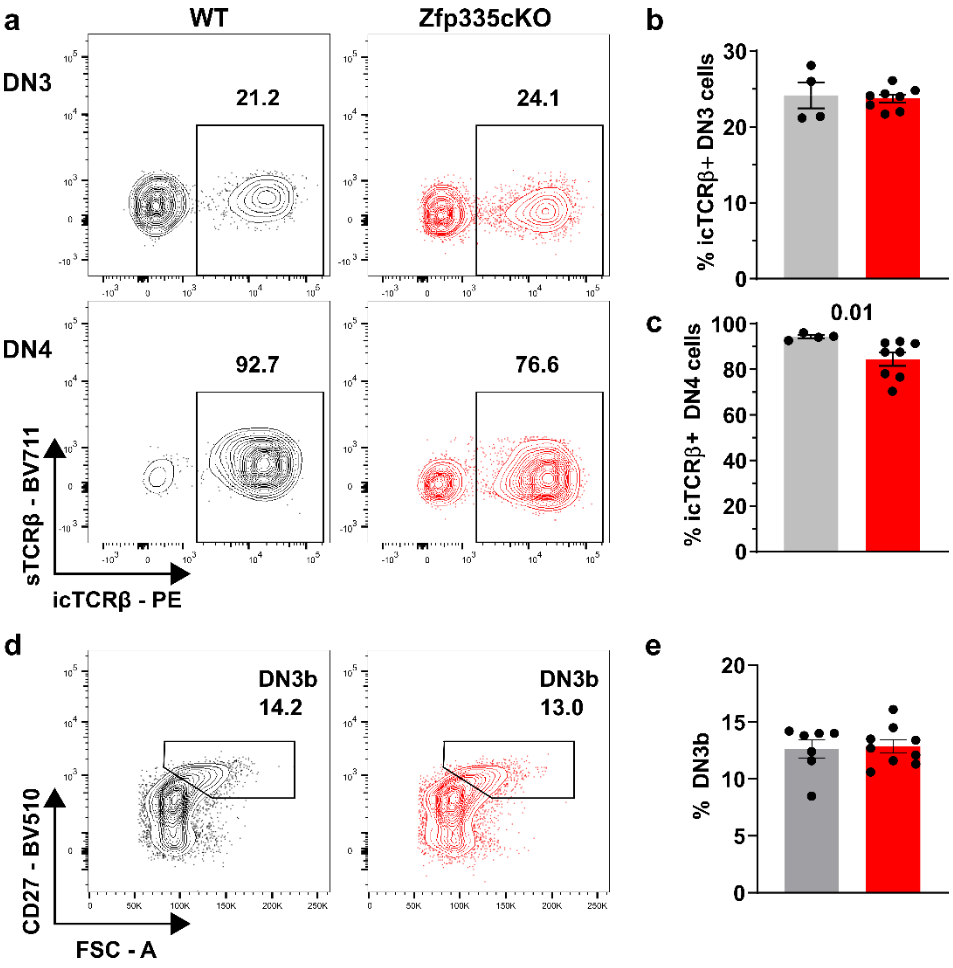

Supplementary Figure 3 – Loss of Zfp335 during DN3 does not impair β-selection. (a) Gating for icTCRβ expression among DN3 ($CD90^{+}$ $TCR\delta^{-}$ $CD4^{-}$ $CD8^{-}$ $sTCR\beta^{-}$ $CD44^{-}$ $CD25^{+}$) or DN4 ($CD90^{+}$ $TCR\delta^{-}$ $CD4^{-}$ $CD8^{-}$ $sTCR\beta^{-}$ $CD44^{-}$ $CD25^{-}$) thymocytes. Frequency of icTCRβ DN3 (b) or DN4 (c) cells among WT (grey, n=4) or Zfp335cKO (red, n=8) male and female mice. (d) Flow cytometric gating for identification of WT or Zfp335cKO DN3b thymocytes pre-gated on total DN3 cells. (e) Quantification of DN3b frequency among WT (n=7) or Zfp335cKO (n=9) DN3 thymocytes from male and female mice. *P*-values determined by Two-way ANOVA with *post hoc* Sidak test (b,c) or two-tailed Mann-Whitney U-Test (e). Plots show mean ± sem. Source data are provided as a Source Data file.

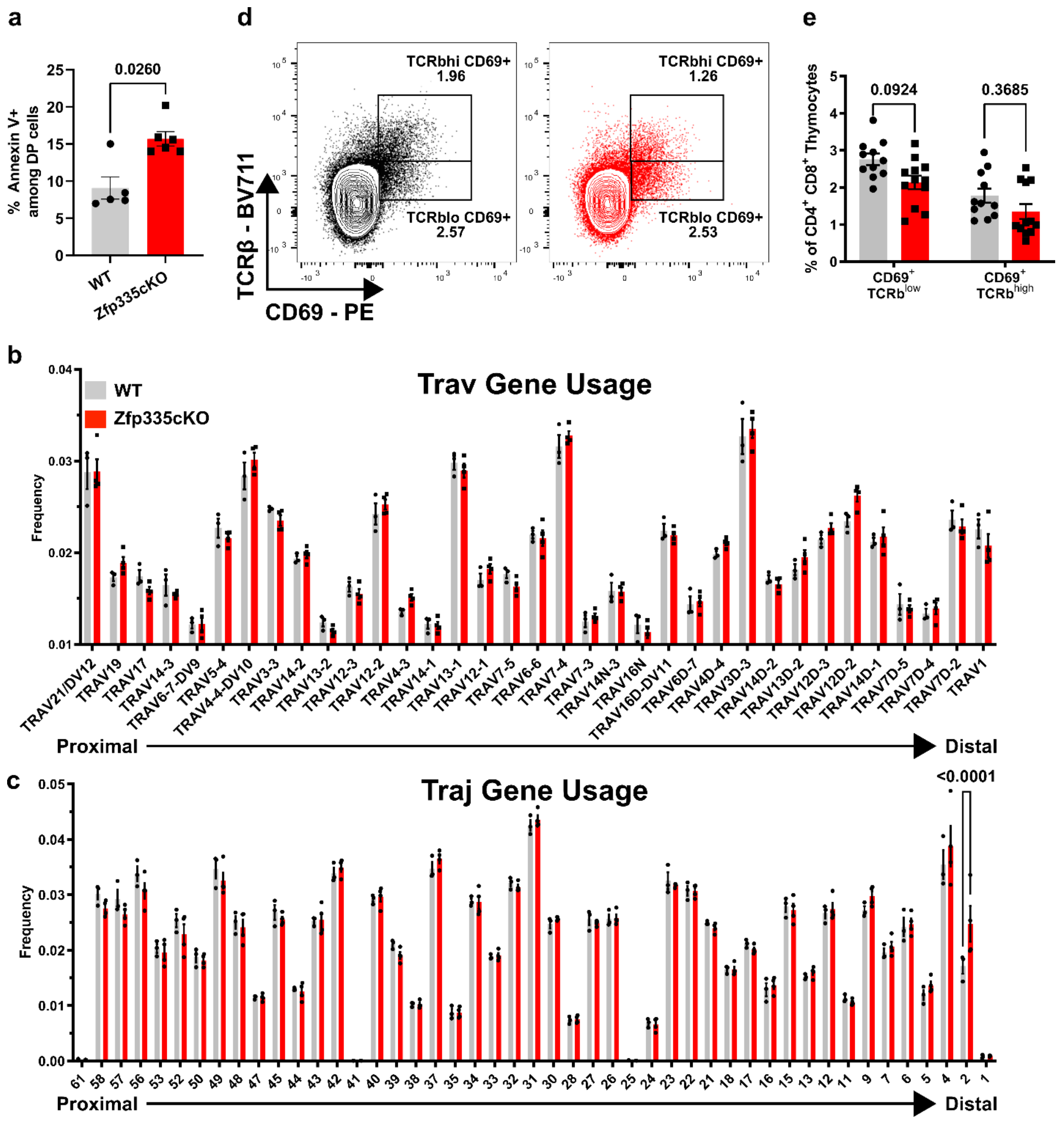

Supplementary Figure 4 – Loss of Zfp335 does not impair DP thymocyte survival. (a) Frequency of Annexin V+ cells among DP thymocytes after culturing DN3/4 thymocytes on OP9-DL1 cells for 3 days (n=5 WT or n=6 Zfp335cKO). Frequency of Trav (b) and Traj (c) gene segment usage in functional *Trac* gene rearrangments in WT (n=3) or Zfp335cKO (n=4) DP thymocytes *ex vivo*. Representative gating (d) and quantification (e) of positive selection among DP thymocytes based on CD69 and TCRβ expression in WT (n=11) or Zfp335cKO (n=12). All data were compiled from experiments using male and female mice. *P*-values determined by two-tailed Mann-Whitney U-Test (a) Two-way ANOVA with *post hoc* Sidak test (b-e) or. Plots show mean ± sem. Source data are provided as a Source Data file.

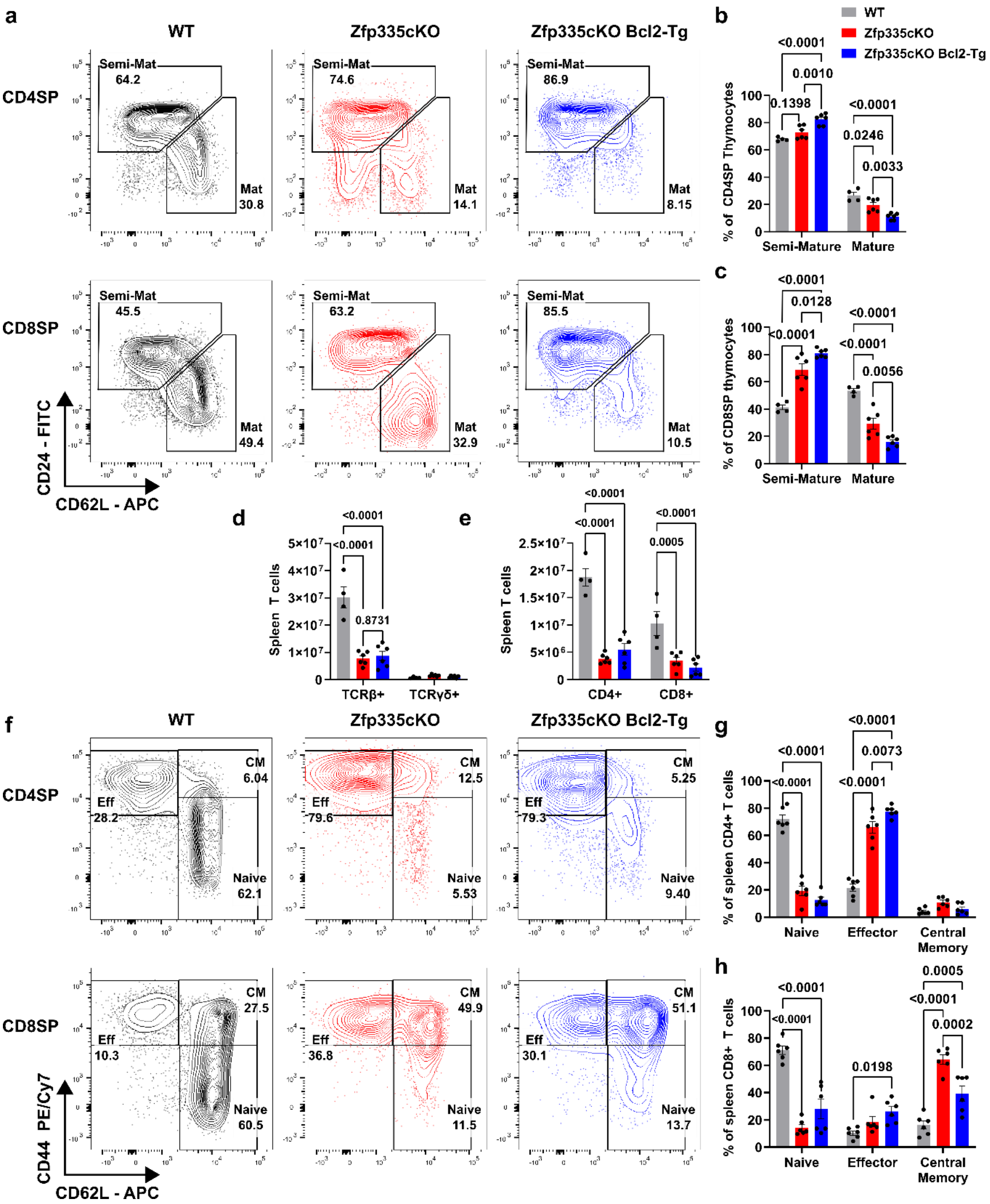

Supplementary Figure 5 – Bcl2 overexpression fails to rescue thymic differentiation defect and peripheral T lymphopenia in Zfp335-deficient mice. Representative gating (a) and quantification of CD4SP (b) or CD8SP (c) thymic maturation. Total splenic TCRβ and TCRγδ (d) T cells. (e) Quantification of total splenic CD4+ or CD8+ TCRβ+ T cells. Representative gating (f) and quantification of splenic CD4+ (g) or CD8+ (h) T cell effector status. n=4 WT, n=6 Zfp335cKO,

n=6 Zfp335cKO Bcl2-Tg male and female mice. Data are compiled from three independent experiments. *P*-values determined by Two-Way ANOVA with *post hoc* Sidak Test. Plots show mean ± sem. Source data are provided as a Source Data file.

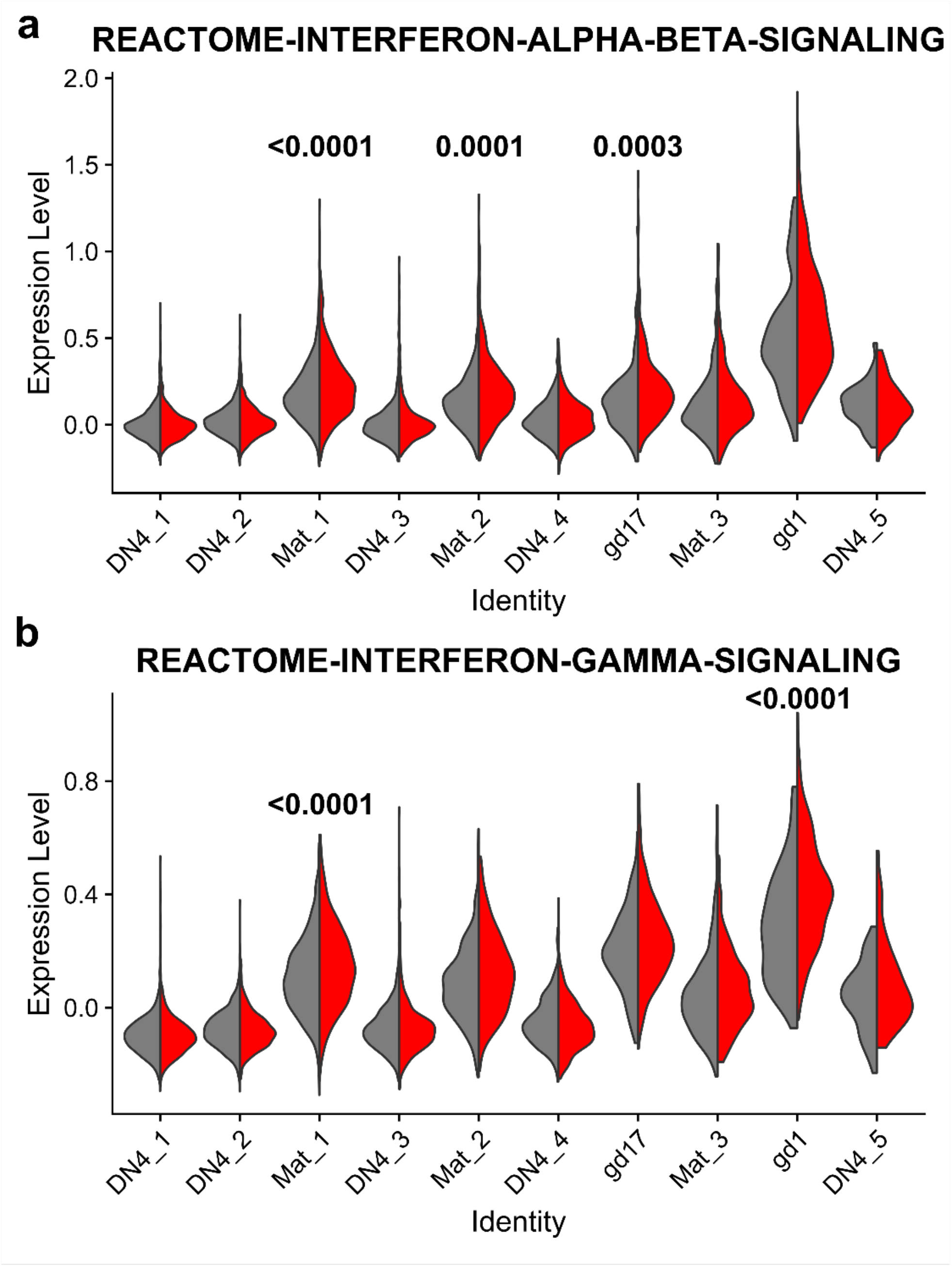

Supplementary Figure 6 – Loss of Zfp335 promotes increased type I and II interferon signaling in γδ and maturing αβ thymocytes. Violin plots showing IFN-I (a) or IFNγ (b) signaling scores for each cluster separated by genotype (WT is grey, Zfp335cKO is red). p-values shown are the adjusted p-values determined by two-sided Wilcoxon Rank Sum tests between WT and Zfp335cKO cells within each cluster. Source data are provided as a Source Data file.

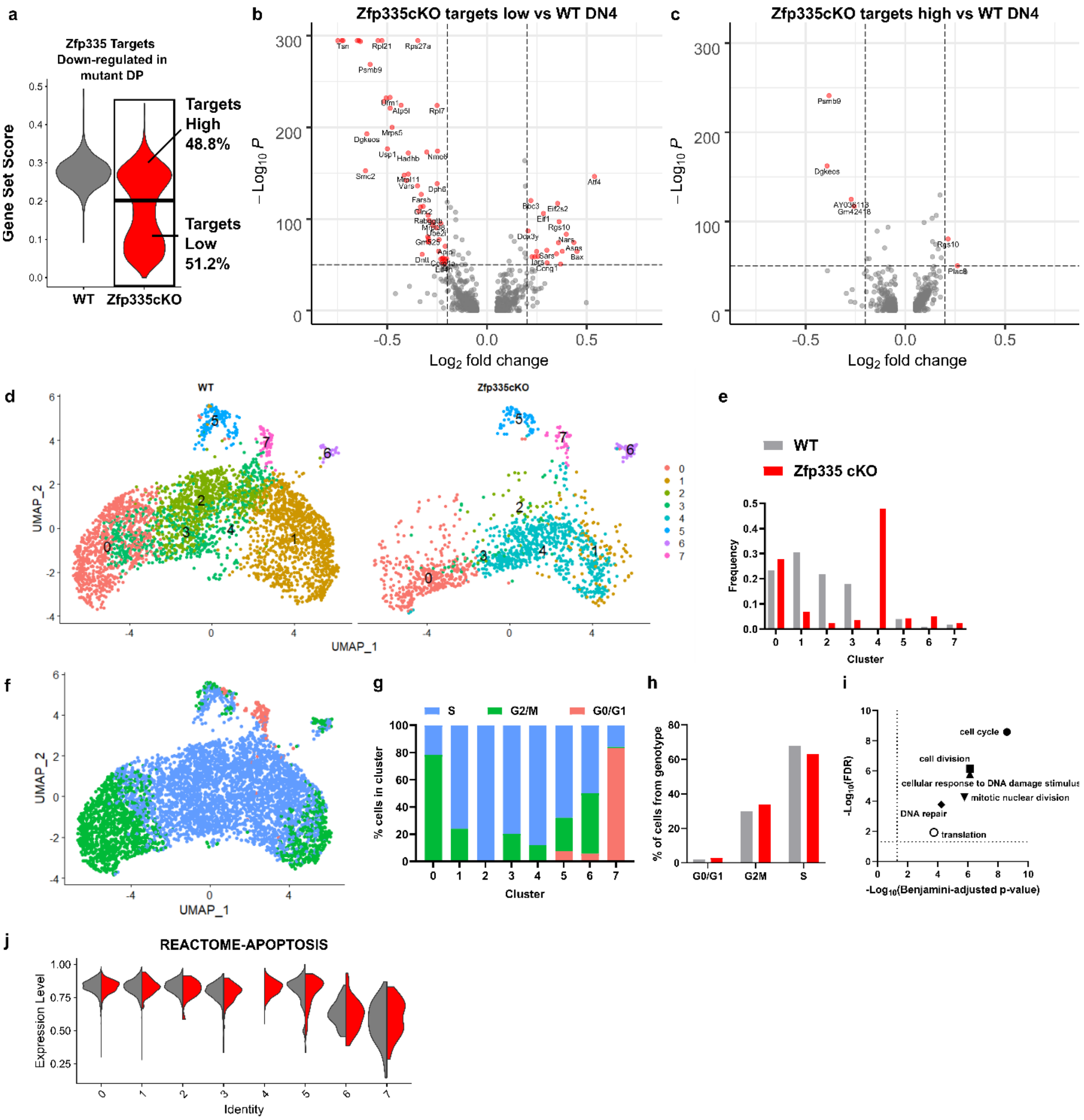

Supplementary Figure 7 – scRNA-seq identifies 'true' Zfp335 mutant DN4 cells. (a) Violin plot of gene set score for Zfp335 target genes down-regulated in mutant DP thymocytes (Fig 1l,m) and cutoff value used to identify 'true' Zfp335 mutant cells with low target score (lower box) and non-mutant cells (upper box). Volcano plots of differentially expressed genes between Zfp335cKO targets low (b) or Zfp335cKO targets high (c) cells compared with WT control. (d) UMAP projections colored by cluster and separated by genotype for WT and true Zfp335 mutant DN4 cells. (e) Frequency of cells found within each cluster. UMAP projection (f) and quantification of cell cycle phase for each cluster (g). (h) Quantification of distribution of cell cycle phase by genotype. (i) GO analysis of top 25 cluster defining genes for each cluster (dashed lines indicate significance cutoff of p<0.05 and FDR<0.05. (j) Violin plot of Reactome-Apoptosis gene signature for WT (grey) or 'true' mutant Zfp335cKO (red) DN4 cells. *P*-values determined by two-sided

Wilcoxon Rank Sum test (b-c) or Fischer’s Exact test with Benjamini-Hochenberg correction (i). Source data are provided as a Source Data file.

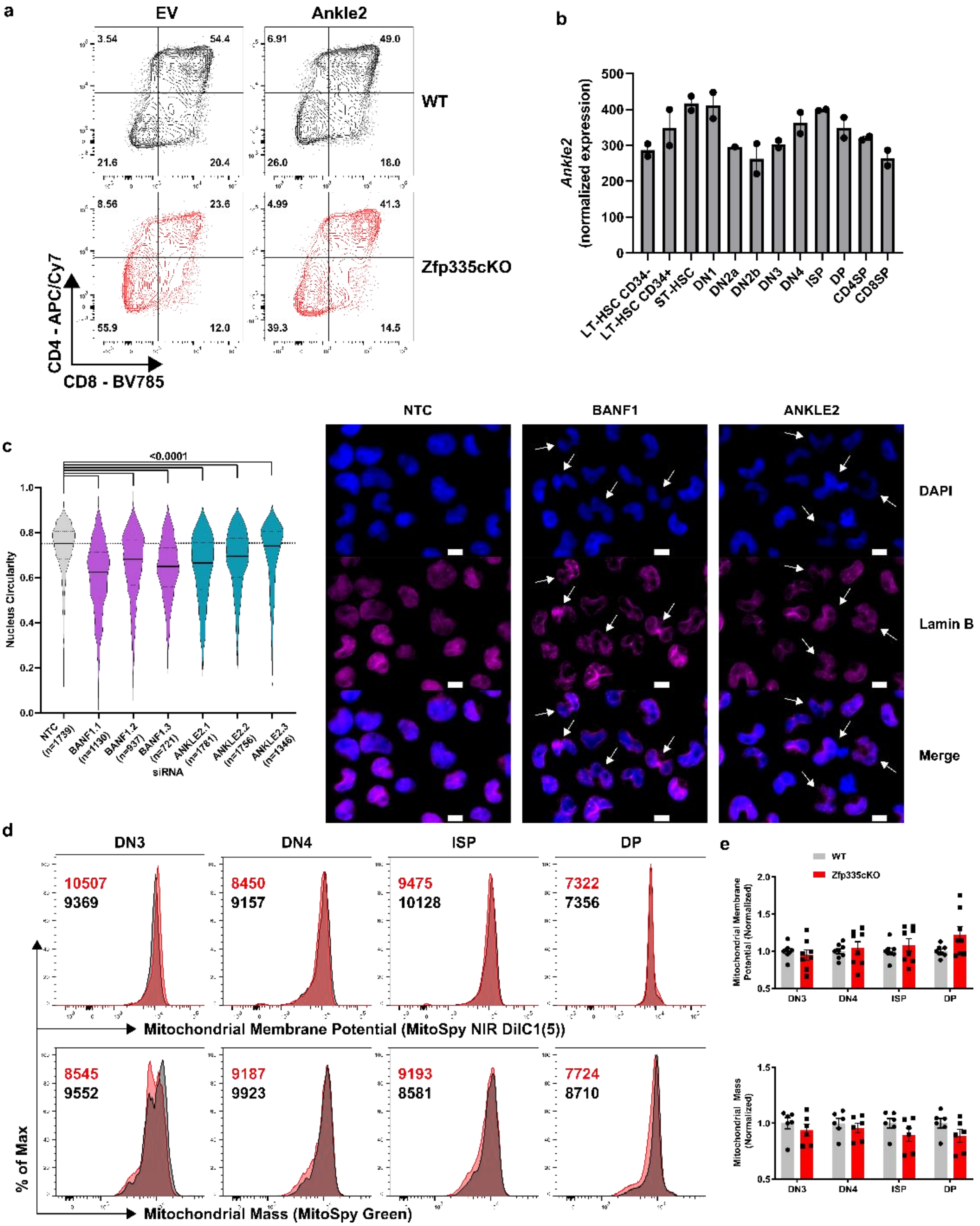

Supplemental Figure 8 – Ankle2/BANF1 control nuclear envelope architecture. (a) Representative gating for developmental progression of EV or Ankle2 retrovirus transduced DN3 thymocytes after 3 days in OP9-DL1 culture (Related to Fig 5h). (b) *Ankle2* transcript expression throughout T cell development determined by RNA-seq (GSE109125). Quantification of nuclear

circularity (c) in Hela cells transfected with non-targeting control (NTC), *BANF1*-, or *ANKLE2*-targeting siRNAs 48 hours post-transfection (left). Representative images DAPI or Lamin B staining of siRNA transfected Hela cells (right). Scale bars are 10µm. Arrows indicate cells with severely disrupted nuclear envelope architecture. Sample sizes for each siRNA are indicated in the violin plot (left). Representative histograms (d) and compiled data (e) for mitochondrial membrane potential (top) or total mitochondrial mass (bottom) in WT (n=8 top or n=6 bottom male and female mice) or Zfp335cKO (n=8 top or n=6 bottom male and female mice) thymocyte populations *ex vivo*. Plots show mean ± sem (a,d) or median (solid line) and interquartile range (dotted lines) (b). Data were compiled from two independent experiments. P-values determined by ordinary One-Way ANOVA with post hoc Dunnett's test (b). Source data are provided as a Source Data file.

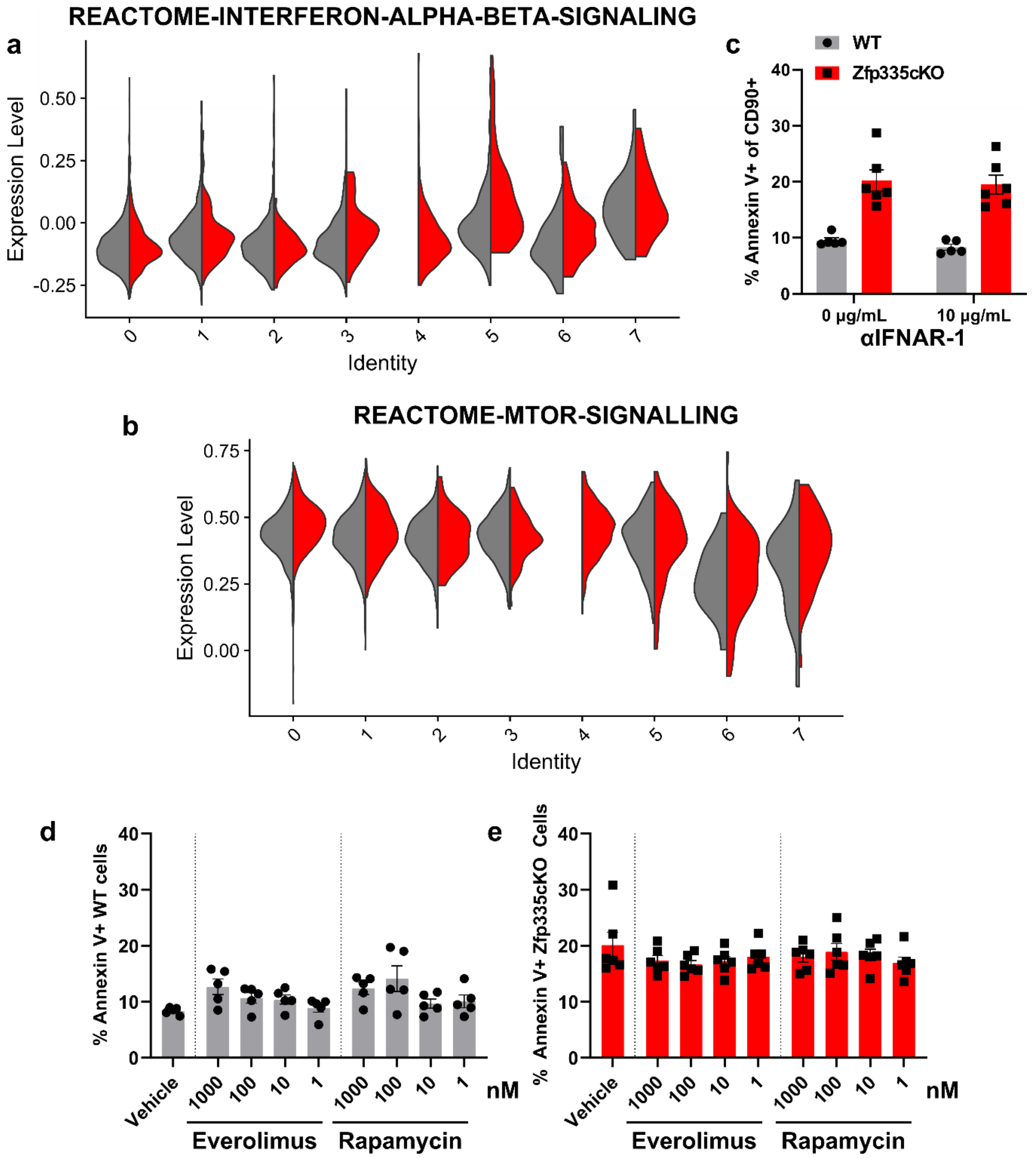

Supplementary Figure 9 – Increased rates of Zfp335cKO apoptosis is not dependent upon mTOR or Type I Interferon signaling. Gene signature scores for Type I Interferon (a) or mTOR (b) signaling among WT (grey) or 'true' mutant Zfp335cKO (red) DN4 thymocytes separated by cluster. (c) Frequency of Annexin V+ cells among total CD90+ thymocytes derived from DN3/4 cells cultured with OP9-DL1 cells for 3 days in the presence (10µg/mL) or absence (0µg/mL) IFNAR-1 blocking antibody. Frequency of Annexin V+ among total CD90+ thymocytes derived from DN3/4 cells cultured with OP9-DL1 cells for 3 days in the presence of 0, 1, 10, 100, or 1000nM Everolimus or Rapamycin from WT (d) or Zfp335cKO mice (e). (c-e) n=5 WT or n=6 Zfp335cKO male and female mice. Graphs show mean ± sem. Data are compiled from one (a,b) or two (c-e) independent experiments. Source data are provided as a Source Data file.

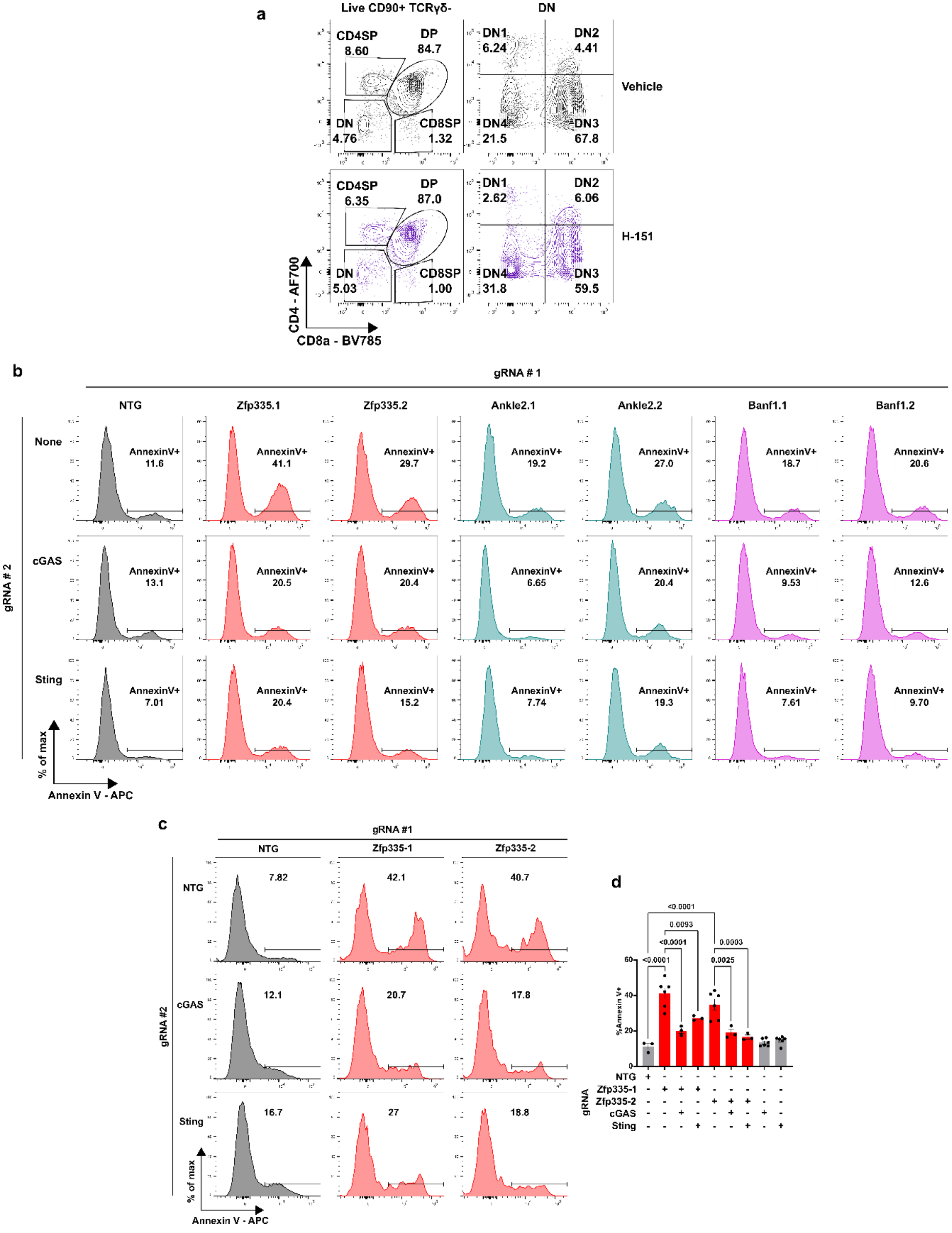

Supplementary Figure 10 – Cas9-mediated Zfp335 deletion drives cGAS/STING-dependent DN4 apoptosis. (a) Representative gating of thymocyte subsets for vehicle (grey) or H-151 (purple)-treated Zfp335cKO mice (related to Fig 7d-h). (b) Representative histograms for apoptosis of DN4 thymocytes derived from *Tcrd*$^{Cre/ERT2}$ *R26*$^{LSL\text{-}Cas9}$ thymocytes transduced with NTG (grey), Zfp335 (red), Ankle2 (teal), or Banf1 (magenta) (gRNA #1) and cGAS or Sting-

targeting (gRNA #2) gRNA expressing retroviruses after 3 days in OP9-DL1 culture (related to Fig 7i). Representative gating (c) and quantification (d) of Annexin V binding among DN4 cells from *R26*$^{LSL\text{-}Cas9}$ *E8*$_{III}$*-cre* DN3/4 thymocytes transduced with indicated gRNA-expressing retroviruses and cultured for three days on OP9-DL1 cells. Grey bars indicate Zfp335 non-targeting control gRNAs. Red bars indicate Zfp335-targeting gRNAs. n=3 male and female mice for each dual gRNA transduction. P-values calculated using One-Way ANOVA with Dunnett's post hoc test. Plots show mean ± sem. Data are representative of (a-b) or compiled from three independent experiments (c-d). Source data are provided as a Source Data file.

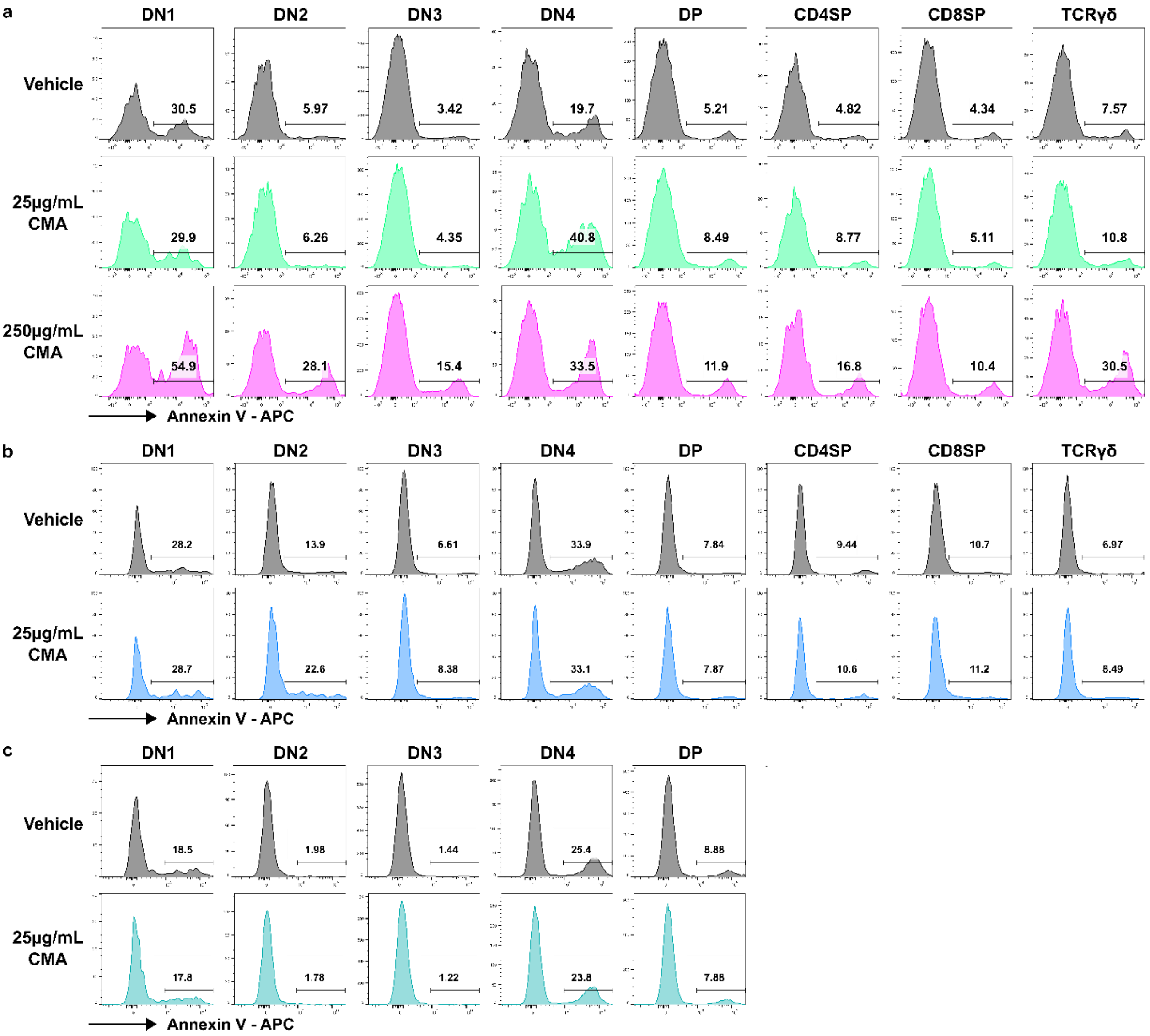

Supplementary Figure 11 – WT DN4 thymocytes exhibit increased sensitivity to STING-mediated apoptosis. Representative gating for apoptosis among thymocyte subsets following overnight stimulation with vehicle or STING agonist (CMA) of WT (a), Zfp335cKO Bcl2Tg (b), or 10 day post-αCD3ε treated *Rag2*$^{-/-}$ thymocytes. Gating is related to Fig 7j-m.

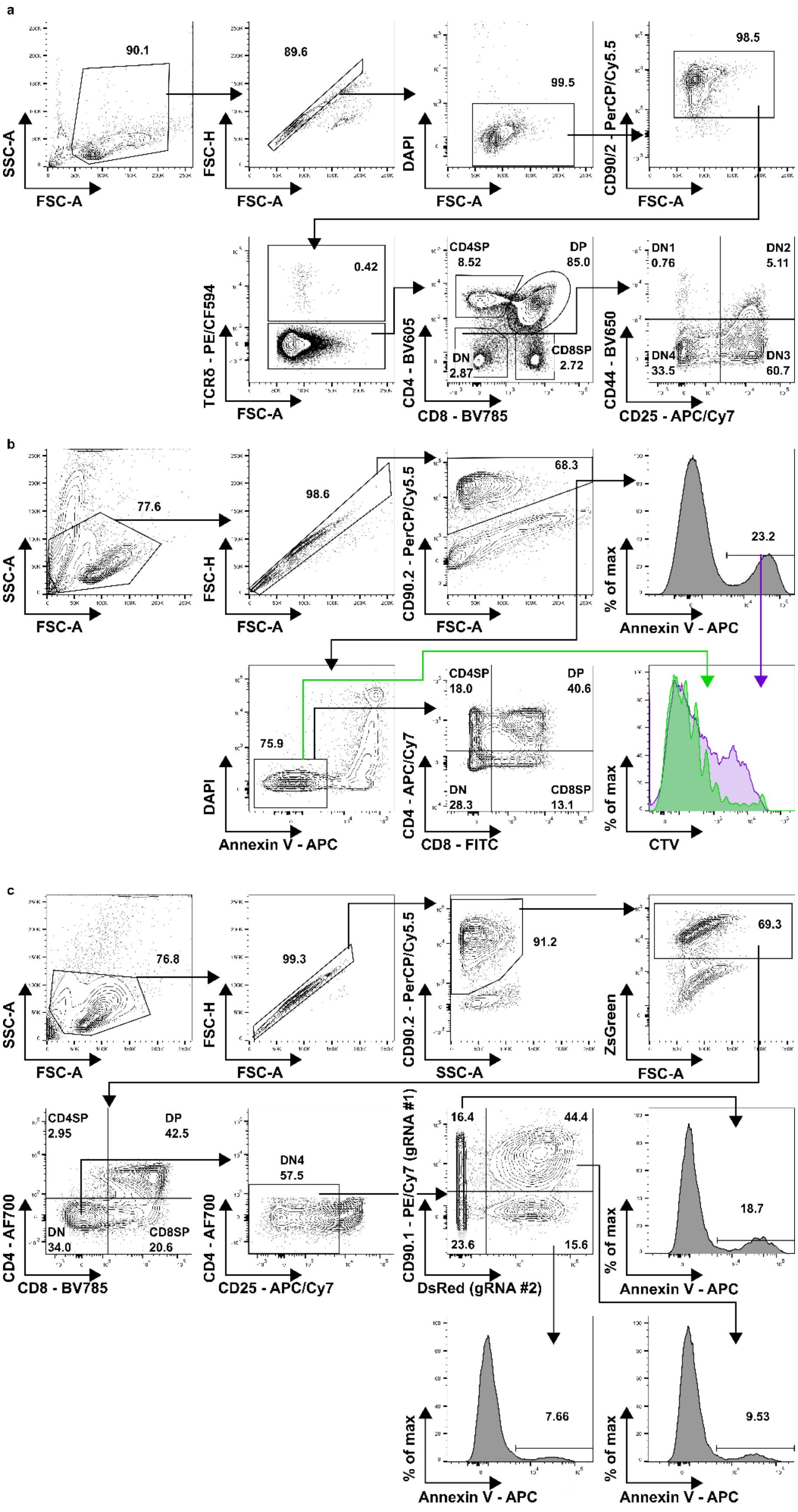
a
90.1
SSC-A
FSC-A
89.6
FSC-H
FSC-A
99.5
DAPI
FSC-A
98.5
CD90/2 - PerCP/Cy5.5
FSC-A
0.42
TCRδ - PE/CF594
FSC-A
CD4SP
8.52
DP
85.0
DN
2.87
CD8SP
2.72
CD4 - BV605
CD8 - BV785
DN1
0.76
DN2
5.11
DN4
33.5
DN3
60.7
CD44 - BV650
CD25 - APC/Cy7
b
77.6
SSC-A
FSC-A
98.6
FSC-H
FSC-A
68.3
CD90.2 - PerCP/Cy5.5
FSC-A
23.2
% of max
Annexin V - APC
75.9
DAPI
Annexin V - APC
CD4SP
18.0
DP
40.6
DN
28.3
CD8SP
13.1
CD4 - APC/Cy7
CD8 - FITC
% of max
CTV
c
76.8
SSC-A
FSC-A
99.3
FSC-H
FSC-A
91.2
CD90.2 - PerCP/Cy5.5
SSC-A
69.3
ZsGreen
FSC-A
CD4SP
2.95
DP
42.5
DN
34.0
CD8SP
20.6
CD4 - AF700
CD8 - BV785
DN4
57.5
CD4 - AF700
CD25 - APC/Cy7
16.4
44.4
23.6
15.6
CD90.1 - PE/Cy7 (gRNA #1)
DsRed (gRNA #2)
18.7
% of max
Annexin V - APC
7.66
% of max
Annexin V - APC
9.53
% of max
Annexin V - APC

Supplementary Figure 12 – Flow cytometry gating schemes. (a) Gating scheme used for ex vivo analyses. First debris was excluded (FSC-A vs SSC-A) followed by doublets, dead and CD90.2$^-$ cells. Thymocyte subsets were then separated based expression of TCRγδ, CD4, CD8, CD44 and CD25. Gating is related to Fig. 1, 3, 6, and 7 and Supp. Fig. 2-5, 8, 11. (b) Gating scheme used for analysis of OP9-DL1 cultures. First debris and OP9-DL1 cells were excluded (FSC-A vs SSC-A) followed by doublets and CD90.2$^-$ cells. Next, cells were separated into viable (Annexin V$^-$ DAPI$^-$) or apoptotic (Annexin V$^+$). Viable cells were assessed for CD4 and CD8 expression or proliferation tracking by CellTrace Violet (CTV) dilution. Gating is related to Fig. 2-7. (c) Gating scheme used for ex vivo CRISPR/Cas9 gene co-targeting followed by OP9-DL1 culture. First debris was excluded (FSC-A vs SSC-A) followed by doublets, OP9-DL1 and CD90.2$^-$ cells. ZsGreen$^+$ (Cas9$^+$) were then gated to identify total DN (CD4$^-$ CD8$^-$) then DN4 (CD25$^{low/-}$). Virally transduced DN4 cells were then separated into single-targeting (CD90.1$^+$ DsRed$^-$ or CD90.1$^-$ DsRed$^+$) or dual-targeting (CD90.1$^+$ DsRed$^+$). Representative plots show targeting with Baf-2 gRNA and cGAS gRNA. Gating is related to Fig. 7 and Supp Fig 10.

Table S1 – Antibodies

Antibody (Clone)	Vendor	Cat. No.; RRID	Dilution
Anti-CD90.2-PerCP/Cy5.5 (30-H12)	Biolegend	105337; RRID: AB_2571944	1:240 or 1:960
Anti-TCRgd-PE/CF594 (GL3)	BD Biosciences	563532; RRID: AB_2661844	1:240
Anti-TCRgd-PE/Cy5 (GL3)	eBiosciences	15-5711-82; RRID: AB_468804	1:240
Anti-TCRgd-Biotin (GL3)	eBiosciences	13-5711-85; RRID: AB_466669	2.5ug/mL
Anti-CD4-BV605 (RM4-5)	Biolegend	100548; RRID: AB_2563054	1:240
Anti-CD4-APC/Cy7 (RM4-5)	Biolegend	100526; RRID: AB_312727	1:240
Anti-CD4-PE/Cy7 (RM4-5)	Biolegend	100528; RRID: AB_312729	1:240
Anti-CD4-FITC (RM4-5)	Biolegend	100510; RRID: AB_312713	1:240
Anti-CD4-Biotin (RM4-5)	Biolegend	100508; RRID: AB_312711	2.5ug/mL
Anti-CD8-BV785 (53-6.7)	Biolegend	100750; RRID: AB_2562610	1:240
Anti-CD8-FITC (53-6.7)	Biolegend	100706; RRID: AB_312745	1:240
Anti-CD8-Biotin (53-6.7)	Biolegend	100704; RRID: AB_312743	2.5ug/mL
Anti-CD44-BV650 (IM7)	Biolegend	103049; RRID: AB_2562600	1:240
Anti-CD44-PE/Cy7 (IM7)	Biolegend	103030; RRID: AB_830787	1:240
Anti-CD44-Biotin (IM7)	Biolegend	103004; RRID: AB_312955	2.5ug/mL
Anti-CD25-PE (PC61)	Biolegend	102008; RRID: AB_312857	1:480
Anti-CD25-APC/Cy7 (PC61)	Biolegend	102026; RRID: AB_830745	1:240
Anti-CD25-Biotin (PC61)	Biolegend	102004; RRID: AB_312853	2.5ug/mL
Anti-CD62L-FITC (MEL-14)	Biolegend	104406; RRID: AB_313093	1:240
Anti-CD62L-APC (MEL-14)	Biolegend	104412; RRID: AB_313099	1:240
Anti-TCRb-BV711 (H57-597)	Biolegend	109243; RRID: AB_2629564	1:240
Anti-TCRb-PE (H57-597)	Biolegend	109208; RRID: AB_313431	1:240
Anti-TCRb-Biotin (H57-597)	Biolegend	109204; RRID: AB_313427	2.5ug/mL
Anti-CD27-BV510 (LG.3A10)	Biolegend	124229; RRID: AB_2565795	1:120
Anti-CD27-APC (LG.3A10)	Biolegend	124212; RRID: AB_2073425	1:120
Annexin V-APC	Biolegend	640941	1:20

Anti-Bcl2-PE/Cy7 (BCL/10C4)	Biolegend	633512; RRID: AB_2565247	1:240
Anti-CD24-FITC (M1/69)	Biolegend	101806; RRID: AB_312839	1:240
Anti-B220-PE/Cy5 (RA3-6B2)	Biolegend	103210; RRID: AB_312995	1:240
Anti-B220-Biotin (RA3-6B2)	Biolegend	103204; RRID: AB_312989	2.5ug/mL
Anti-CD11b-PE/Cy5 (M1/70)	Biolegend	101210; RRID: AB_312793	1:240
Anti-CD11b-Biotin (M1/70)	Biolegend	101204; RRID: AB_312787	2.5ug/mL
Anti-CD11c-PE/Cy5 (N418)	Biolegend	117316; RRID: AB_493566	1:240
Anti-CD11c-Biotin (N418)	Biolegend	117304; RRID: AB_313773	2.5ug/mL
Anti-CD19-PE/Cy5 (6D5)	Biolegend	115510; RRID: AB_313645	1:240
Anti-CD19-Biotin (6D5)	Biolegend	115504; RRID: AB_313639	2.5ug/mL
Anti-Ly-6G/Ly-6C (GR-1)-PE/Cy5 (RB6-8C5)	Biolegend	108410; RRID: AB_313375	1:240
Anti-Ly-6G/Ly-6C (GR-1)-Biotin (RB6-8C5)	Biolegend	108404; RRID: AB_313369	2.5ug/mL
Anti-NK1.1-PE/Cy5 (PK136)	Biolegend	108716; RRID: AB_493590	1:240
Anti-NK1.1-Biotin (PK136)	Biolegend	108704; RRID: AB_313391	2.5ug/mL
Anti-TER119-PE/Cy5 (TER-119)	Biolegend	116210; RRID: AB_313711	1:240
Anti-TER110-Biotin (TER-119)	Biolegend	116204; RRID: AB_313705	2.5ug/mL
Anti-CD117(c-kit)-Biotin (2B8)	Biolegend	105804; RRID: AB_313213	2.5ug/mL
Anti-CD16/32-Biotin (2.4G2)	Tonbo Biosciences	30-0161-U500; RRID: AB_	2.5ug/mL
Anti-CD90.1-PE (OX7)	Biolegend	202524; RRID: AB_1595524	1:480
Anti-CD90.1-PE/Cy7 (OX7)	Biolegend	202518; RRID: AB_1659223	1:240
Anti-Lamin B-Purified (10H34L18)	Thermo Fisher Scientific	702972; RRID: AB_2784553	1:100
Anti-IFNAR-1 UltraLEAF Purified (MAR1-5A3)	Biolegend	127322; RRID: AB_11149116	10ug/mL
Anti-phosphoserine (M380B)	Biolegend	944101; RRID: AB_2890869	1:100
Anti-CD90.2-AF647 (30-H12)	Biolegend	105318; RRID: AB_492888	1:250
Anti-phospho IRF3 (polyclonal)	ThermoFisher Scientific	PA5-36775; RRID: AB_2553722	1:250
Anti-phospho STING (polyclonal)	ThermoFisher Scientific	PA5-105674; RRID: AB_2817102	1:250
Anti-phospho TBK1-PE (D52C2)	Cell Signaling Technologies	13498; RRID: AB_2798237	1:100

Anti-Banf1 (polyclonal)	ThermoFisher Scientific	PA5-20329; RRID: AB_11152795	1:100
Donkey anti-Rabbit IgG (H+L)-AlexaFluor 488	ThermoFisher Scientific	A21206; RRID: AB_2535792	1:500
Goat anti-Mouse IgG (H+L)-AlexaFluor 488	ThermoFisher Scientific	A11029; RRID: AB_2534088	1:500
Goat anti-Rabbit IgG (H+L)-Alexa Fluor 647	Thermo Fisher Scientific	A32733; RRID: AB_2633282	1:500

Table S2. Primer sequences (Related to Figures 3, 5, 7, S1, S4 and S10)

Primer	Gene	Sequence	Purpose
Zfp335-F	*Zfp335*	CATGTGGTTTCTGGGAAAAACT	$Zfp335^{fl/fl}$ recombination
Zfp335-ex2F	*Zfp335*	GACCGTCCCAGGATTAAC	$Zfp335^{fl/fl}$ recombination
Zfp335-ex2R	*Zfp335*	CTCTTCCATGATCACTACCC	$Zfp335^{fl/fl}$ recombination
FseI-Kz-Bcl2-F	Bcl2	AA<u>GGCCGGCC</u>GCCGCCACC*ATGGCGCAAGCCGGGA*	Ai6-Bcl2 cloning
SfiI-Bcl2-R	Bcl2	AA<u>GGCCTGTGTGGCC</u>*TCACTTGTGGCCCAGGTATGCAC*	Ai6-Bcl2 cloning
Ankle2-NEB-F	Ankle2	AGATCTCTCGAGATCGATGC*ATGCTGTGGCAGCGGCTG*	MSCV-Ankle2-IRES-Thy1.1 cloning
Ankle2-NEB-R	Ankle2	TATCGGGAATTATCGATGCA*TCACAGAGAAATGAAGTCCAGGGC*	MSCV-Ankle2-IRES-Thy1.1 cloning
mmGapdh-F	Gapdh	GTCATCCCAGAGCTGAACG	RT-qPCR
mmGapdh-R	Gapdh	TCATACTTGGCAGGTTTCTCC	RT-qPCR
mmAnkle2-F	Ankle2	TTAAACCGGGACCCTTTGAT	RT-qPCR
mmAnkle2-R	Ankle2	ATATGAGGATGGCCCTGTGA	RT-qPCR
mmZfp335-F	Zfp335	CCAGGAACAGACAGTGACCAA	RT-qPCR
mmZfp335-R	Zfp335	CCTTCCTGGACCTGGATATGA	RT-qPCR
mmBax-F	Bax	TGAAGACAGGGGCCTTTTTG	RT-qPCR
mmBax-R	Bax	AATTCGCCGGAGACACTCG	RT-qPCR
Zfp335_iT1	*Zfp335* Promoter	ttgtttGACCTCGTCGATGCCGGAGT	CRISPRi
Zfp335_iT2	*Zfp335* Promoter	ttgtttGCTGTGTCGCTCTCCGACTC	CRISPRi
Zfp335_iT3	*Zfp335* Promoter	ttgtttAGGCTCAGGTTAGCGGCAGC	CRISPRi
Zfp335_iT4	*Zfp335* Promoter	ttgtttCTCAGGTTAGCGGCAGCCGG	CRISPRi
Zfp335_iT5	*Zfp335* Promoter	ttgtttCTGCCGCTAACCTGAGCCTC	CRISPRi
Zfp335_iB1	*Zfp335* Promoter	aaacACTCCGGCATCGACGAGGTCaa	CRISPRi
Zfp335_iB2	*Zfp335* Promoter	aaacGAGTCGGAGAGCGACACAGCaa	CRISPRi
Zfp335_iB3	*Zfp335* Promoter	aaacGCTGCCGCTAACCTGAGCCTaa	CRISPRi
Zfp335_iB4	*Zfp335* Promoter	aaacCCGGCTGCCGCTAACCTGAGaa	CRISPRi
Zfp335_iB5	*Zfp335* Promoter	aaacGAGGCTCAGGTTAGCGGCAGaa	CRISPRi

Zfp335_kT1	*Zfp335* exon 7	ttgtttGTACCCCGAGACCTCGACGG	*Ex vivo* CRISPR KO
Zfp335_kB1	*Zfp335* exon 7	aaacCCGTCGAGGTCTCGGGGTACaa	*Ex vivo* CRISPR KO
Zfp335_kT2	*Zfp335* exon 16	ttgtttACCACAATCATCTACCAGCA	*Ex vivo* CRISPR KO
Zfp335_kB2	*Zfp335* exon 16	aaacTGCTGGTAGATGATTGTGGTaa	*Ex vivo* CRISPR KO
Ankle2_kT1	*Ankle2* exon 4	ttgtttGCGGAAAGCTGTCGAAAACG	*Ex vivo* CRISPR KO
Ankle2_kB1	*Ankle2* exon 4	aaacCGTTTTCGACAGCTTTCCGCaa	*Ex vivo* CRISPR KO
Ankle2_kT2	*Ankle2* exon10	ttgtttGGGAGCTAGCTCATGAGCTG	*Ex vivo* CRISPR KO
Ankle2_kB2	*Ankle2* exon 10	aaacCAGCTCATGAGCTAGCTCCCaa	*Ex vivo* CRISPR KO
Banf1_kT1	*Banf1* exon 2	ttgtttTTGGTGACGTCCTGAGCAAG	*Ex vivo* CRISPR KO
Banf1_kB1	*Banf1* exon 2	aaacCTTGCTCAGGACGTCACCAAaa	*Ex vivo* CRISPR KO
Banf1_kT2	*Banf1* exon 2	ttgtttACTTCGTGGCAGAGCCCATG	*Ex vivo* CRISPR KO
Banf1_kB2	*Banf1* exon 2	aaacCATGGGCTCTGCCACGAAGTaa	*Ex vivo* CRISPR KO
Mb21d1_kT	*Mb21d1* exon 3	ttgtttTGATAAGAAGTGTTACAGCA	*Ex vivo* CRISPR KO
Mb21d1_kB	*Mb21d1* exon 3	aaacTGCTGTAACACTTCTTATCAaa	*Ex vivo* CRISPR KO
Tmem173_kT	*Tmem173* exon 6	ttgtttCTACATAACAACATGCTCAG	*Ex vivo* CRISPR KO
Tmem173_kB	*Tmem173* exon 6	aaacCTGAGCATGTTGTTATGTAGaa	*Ex vivo* CRISPR KO
DsRed-NEB-F	dsRed Express II	CCGACCTCTCTCCCCAGGGGATGGATAGCACTGAGAAC	Replace Thy1.1 with dsRed in CRISPR KO vectors
DsRed-NEB-R	dsRed Express II	ATAAAATCTTTTATTTTATCGCTACTGGAACAGGTGGTG	Replace Thy1.1 with dsRed in CRISPR KO vectors
Trac-RT	TCRa C region	TTTCGGCACATTGATTTG	TCRa Rep-Seq (RT)
SmartNNNa	N/A	AAGCAGUGGTAUCAACGCAGAGUNNNNUNNNNUNNNNUCTTrGrGrG	TCRa Rep-Seq (RT)

Tcra-n1R	TCRa C region	ATTGGGCAGCCCTGATTGGTGCTGTCCTGAGACCGAG	TCRa Rep-Seq PCR1
M1SS	TSO	AAGCAGTGGTATCAACGCA	TCRa Rep-seq PCR1
M1S-B1	M1SS	NNNNTTGACTCAGTGGTATCAACGCAG	TCRa Rep-seq PCR2 (barcoding)
Z-B1	Tcra-n1R	NNNNGGCCACATTGGGCAGCCCTGATT	TCRa Rep-seq PCR2 (barcoding)
M1S-B2	M1SS	NNNNGGAACTCAGTGGTATCAACGCAG	TCRa Rep-seq PCR2 (barcoding)
Z-B2	Tcra-n1R	NNNNCGAAACATTGGGCAGCCCTGATT	TCRa Rep-seq PCR2 (barcoding)
M1S-B3	M1SS	NNNNTGACATCAGTGGTATCAACGCAG	TCRa Rep-seq PCR2 (barcoding)
Z-B3	Tcra-n1R	NNNNCGTACGATTGGGCAGCCCTGATT	TCRa Rep-seq PCR2 (barcoding)
M1S-B4	M1SS	NNNNGGACGGCAGTGGTATCAACGCAG	TCRa Rep-seq PCR2 (barcoding)
Z-B4	Tcra-n1R	NNNNCCACTCATTGGGCAGCCCTGATT	TCRa Rep-seq PCR2 (barcoding)
M1S-B5	M1SS	NNNNGCGGACCAGTGGTATCAACGCAG	TCRa Rep-seq PCR2 (barcoding)
Z-B5	Tcra-n1R	NNNNATCAGTATTGGGCAGCCCTGATT	TCRa Rep-seq PCR2 (barcoding)
M1S-B6	M1SS	NNNNTTTCACCAGTGGTATCAACGCAG	TCRa Rep-seq PCR2 (barcoding)
Z-B6	Tcra-n1R	NNNNAGGAATATTGGGCAGCCCTGATT	TCRa Rep-seq PCR2 (barcoding)
M1S-B7	M1SS	NNNNCTCTACCAGTGGTATCAACGCAG	TCRa Rep-seq PCR2 (barcoding)
Z-B7	Tcra-n1R	NNNNCTTTTGATTGGGCAGCCCTGATT	TCRa Rep-seq PCR2 (barcoding)
